# Depression, anxiety, and associated factors in patients with diabetes: evidence from the anxiety, depression, and personality traits in diabetes mellitus (ADAPT-DM) study

**DOI:** 10.1186/s12888-020-02615-y

**Published:** 2020-05-12

**Authors:** Luke Sy-Cherng Woon, Hatta Bin Sidi, Arun Ravindran, Paula Junggar Gosse, Roslyn Laurie Mainland, Emily Samantha Kaunismaa, Nurul Hazwani Hatta, Puteri Arnawati, Amelia Yasmin Zulkifli, Norlaila Mustafa, Mohammad Farris Iman Leong Bin Abdullah

**Affiliations:** 1 Department of Psychiatry, Universiti Kebangsaan Malaysia Medical Center (UKMMC), 56000 Cheras, Kuala Lumpur, Malaysia; 2 Faculty of Medicine, University of Toronto, 1 King’s College Circle, Toronto, ON M5S 1A8 Canada; 3 Faculty of Medicine, Universiti Kebangsaan Malaysia Medical Center (UKMMC), 56000 Cheras, Kuala Lumpur, Malaysia; 4 Lifestyle Science Cluster, Advanced Medical and Dental Institute, Universiti Sains Malaysia, SAINS@BERTAM, 13200 Kepala Batas, Pulau Pinang Malaysia

**Keywords:** Anxiety, Depression, Diabetes mellitus, Personality traits, Quality of life

## Abstract

**Background:**

Depression and anxiety are common psychiatric complications affecting patients with diabetes mellitus. However, data on the prevalence of depression, anxiety, and associated factors among Malaysian diabetic patients is scarce. The Anxiety, Depression, and Personality Traits in Diabetes Mellitus (ADAPT-DM) study aimed to determine the prevalence of depression and anxiety, and their associated factors in the Malaysian diabetic population.

**Methods:**

This cross-sectional study recruited 300 diabetic patients via convenience sampling from the Endocrine outpatient clinic of Universiti Kebangsaan Malaysia Medical Centre, a tertiary referral healthcare facility in Kuala Lumpur. Socio-demographic characteristics and clinical history were obtained from each participant. The Generalised Anxiety Disorder-7 (GAD-7) was administered to assess anxiety symptoms, the Beck Depression Inventory (BDI) to assess depressive symptoms, the Big Five Inventory (BFI) to evaluate personality traits, and the World Health Organization Quality of Life-BREF (WHOQOL-BREF) to measure quality of life (QOL). Stepwise multiple logistic regression analyses were performed to determine the association between various factors, and depression and anxiety.

**Results:**

The prevalence of depression was 20% (*n* = 60) while anxiety was 9% (*n* = 27). Co-morbid depression (adjusted odds ratio [OR] = 9.89, 95% confidence interval [CI] = 2.63–37.14, *p* = 0.001) and neuroticism (adjusted OR = 11.66, 95% CI = 2.69–50.47, *p* = 0.001) increased the odds of developing anxiety, while conscientiousness (adjusted OR = 0.45, 95% CI = 0.23–0.80, *p* = 0.004) and greater psychological-related QOL (adjusted OR = 0.47, 95% CI = 0.29–0.75, *p* = 0.002) were protective. Co-morbid anxiety (adjusted OR = 19.83, 95% CI = 5.63–69.92, *p* <  0.001) increased the odds of depression, while older age (adjusted OR = 0.96, 95% CI = 0.93–0.98, *p* = 0.002), social relationship-related QOL (adjusted OR = 0.84, 95% CI = 0.71–.0.99, *p* = 0.047), and physical health-related QOL (adjusted OR = 0.69, 95% CI = 0.58–0.83, *p* <  0.001) were protective.

**Conclusions:**

The study findings signify the need to screen for co-morbid depression and anxiety, as well as personality traits and QOL, and to include psychosocial interventions when planning a multidisciplinary approach to managing diabetes.

## Background

Diabetes mellitus is a heterogeneous disorder that can result in severe morbidity with substantial emotional impact. The International Diabetes Federation (IDF) has estimated that approximately 425 million adults between the age of 2–79 years were afflicted with diabetes in 2017, and this number is projected to increase up to 629 million by 2045 [1]. Although diabetes is an international health crisis, its prevalence is increasing more rapidly in lower- and middle-income countries [2].

In recent decades, research has focused on the mental health comorbidities associated with diabetes. The occurrence of anxiety and depressive disorders can be as high as two-fold greater in individuals suffering from diabetes. These mood disorders are linked to suboptimal diabetic self-care, unhealthy behaviours, elevated HbA1c, and other sub-optimal metabolic indicators [3–7].

Evidence suggests a bidirectional relationship between diabetes mellitus, and anxiety and depressive disorders. Patients with anxiety symptoms may be at increased risk of developing type 2 diabetes and vice versa [7]. Various factors may contribute to the development of anxiety disorders among patients with diabetes, including personal and family history, stressful life events, substance use, and physical illness [7]. It is possible that diabetes and depression stem from similar or shared aetiologies, or the existence of one condition may increase the prevalence of the other [8]. Possible risk factors that may contribute to the development of depression in diabetic patients are personal and family history, stressful life events, domestic violence, physical illnesses, and clinical factors [8–11].

The relationship between diabetes and psychiatric disorders is still not fully understood.

Personality traits and quality of life (QOL) may contribute to the development and severity of psychiatric disorders in patients with diabetes, but this has not been comprehensively studied. Furthermore, data on the prevalence of depression and anxiety among Malaysian diabetic patients are scarce. In fact, much of the research on diabetes as it relates to personality and mood disorders has been conducted in higher-income countries, despite the strong need to understand these relationships in lower- and middle-income countries where individuals may face additional barriers to care [12]. Hence, the Anxiety, Depression, and Personality Traits in Diabetes Mellitus (ADAPT-DM) study was conducted to examine the prevalence of depression and anxiety among Malaysian diabetic patients, and to investigate their associated socio-demographic characteristics, personality traits, and QOL.

## Methods

### Study design and participants

The ADAPT-DM study used a cross-sectional design and participants were recruited over a period of 2 months at the Endocrine outpatient clinic of Universiti Kebangsaan Malaysia Medical Centre (UKMMC), a tertiary referral centre in Kuala Lumpur, Malaysia. Approval was obtained from the Human Ethics Committee of the Faculty of Medicine, Universiti Kebangsaan Malaysia (UKM FPR.SPI 800–2/28/166/FF-2019-342). Sample size was calculated based on previous estimates of the prevalence of depression and generalized anxiety disorder in patients with diabetes, which are 20 and 14% respectively [13, 14]. The sample size required was 234 subjects. Study participants were recruited via convenience sampling, in which those who attended the Endocrine clinic as outpatients and interested in.

participating were provided with detailed explanations of the study by the researchers. Then, they were screened for inclusion criteria, such as (1) being 18 years and above and (2) having a confirmed diagnosis of type 1, type 2, or gestational diabetes mellitus. Patients with impaired mental capacity, such as those with psychotic features or cognitive impairment, were excluded from the study. The eligible participants were allowed to participate in the study if verbal and written informed consent was given. All participants who were found to have depression and anxiety disorders were referred to Department of Psychiatry, UKMMC for further assessment.

### Measuring tools

The participants completed a questionnaire which collected data on demographic, social, and clinical characteristics. The demographic variables included age, gender, marital status, ethnicity, education level, employment status, household income, and religion. The social variables included perceived level of social support, smoking, alcohol use, and recreational drug use. Clinical variables included medical history, diabetes history (onset, type, and use of insulin therapy), body mass index (BMI), and self-perceived management of diabetes (assessed using a five-point Likert scale). Information provided from the questionnaire was supplemented by a review of patient medical records where applicable. In addition, the participants were administered the seven-item Generalised Anxiety Disorder scale (GAD-7) to assess the prevalence of anxiety, Beck Depression Inventory-II (BDI-II) to assess the prevalence of depression, Big Five Inventory (BFI) to assess personality traits, and the World Health Organization Quality of Life-BREF (WHOQOL-BREF) to measure quality of life (QOL).

#### Seven-item generalised anxiety disorder scale (GAD-7)

The GAD-7 is a self-reported questionnaire designed to screen for generalized anxiety disorder (GAD). It consists of seven items, with each item being assessed using a Likert scale of 0 to 3. Hence, its total score ranges from 0 to 21. The GAD-7 has been found to be a good case-finding instrument for GAD. Participants with scores ≥8 are classified as having GAD. The GAD-7 has proven to be reliable, with a sensitivity of 92% and specificity of 76% at cut-off point of ≥8 [15]. The Malay version of the GAD-7 used in this study has been proven to be reliable, with a sensitivity of 76% and specificity of 94% [16].

#### Beck depression inventory-II (BDI-II)

The BDI-II is a self-reported questionnaire commonly used to screen for and assess the severity of depression. It is comprised of items that are related to depressive symptoms. It is made up of 21 items and each scored from 0 to 3. A score of 10 to 16 indicates mild depression, a score of 17 to 29 indicates moderate depression, and a score between 30 and 63 indicates severe depression [17]. The BDI-II has been proven to have good internal consistency, with Cronbach’s α of 0.91. The Malay version of the BDI-II has also been deemed to have good internal consistency (Cronbach’s α = 0.71 to 0.91) and excellent psychometric properties [18].

#### Big five inventory (BFI)

The BFI is a short instrument that assesses personality traits based on the Five Factor model. The BFI includes 44 items divided into five subscales: extraversion, agreeableness, conscientiousness, neuroticism, and openness. Each item is scored on a five-point Likert scale ranging from 0 (strongly agree) to 4 (strongly disagree) [19]. The Malay version of the BFI has been proven to have good internal consistency, as well as good convergent and discriminant validity [20]. There is no study which investigate the norms of the BFI subscale scores in the general population. Nevertheless, a study with a large sample of American adults aged 21 to 60 years (*n* = 132,515) indicated that the range of the mean of the BFI subscale scores were as follow: 3.10 (SD = 0.85) to 3.31 (SD = 0.90) (extraversion), 3.64 (SD = 0.72) to 4.01 (SD = 0.67) (agreeableness), 3.45 (SD = 0.73) to 3.93 (SD = 0.73) (conscientiousness), 2.92 (SD = 0.99) to 3.32 (SD = 0.82) (neuroticism), and 3.79 (SD = 0.73) to 3.96 (SD = 0.66) (openness) [21].

#### World Health Organization quality of life-BREF (WHOQOL-BREF)

The WHOQOL-BREF is a self-reported questionnaire that assesses quality of life. It is comprised of 26 items. Items 1 and 2 assess overall quality of life, while the remaining items are grouped into four categories that evaluate different domains: physical health, psychological, social relationships, and environmental quality of life. Each item is scored on a Likert scale ranging from 1 to 5. The WHOQOL-BREF has good psychometric properties and has been proven to be a valid and reliable alternative to the WHOQOL-100 for measuring quality of life [22]. The Malay version of the WHOQOL-BREF has also demonstrated excellent psychometric properties with internal consistency (Cronbach’s α) of 0.89 [23]. The general norms for the WHOQOL-BREF domains were 73.5 (SD = 18.1) for physical health quality of life, 70.6 (SD = 14.0) for psychological quality of life, 71.5 (SD = 18.2) for social relationships quality of life, and 75.1 (SD = 13.0) for environmental quality of life [24].

### Data analysis

Statistical analysis was carried out using the Statistical Package for Social Science (SPSS) (version 20.0; IBM, Armonk, NY). Descriptive statistics were computed in which categorical variables were reported in frequency and percentage, and continuous variables reported in median and interquartile range (IQR). The continuous variables were not normally distributed as demonstrated by the Kolmogorov-Smirnov test (*p* <  0.05). The missing data was resolved with mode imputation as the variables with missing data were all categorical variables. Initially, univariate logistic regression was performed to detect any individual association between demographic, social, and clinical characteristics, the BFI and the WHOQOL-BREF scores (independent variables) and anxiety and depression (0 = absence of anxiety/depression, 1 = presence of anxiety/depression) (dependent variable). Variables with *p* <  0.25 were entered into a stepwise multiple logistic regression model as independent variables to determine the factors which significantly predict occurrence of anxiety and depression among participants. The goodness-of-fit of the stepwise multiple logistic regression model was assessed with the Hosmer-Lemeshow goodness-of-fit test, in which *p* > 0.05 was considered as good model fit. The ability of the model to discriminate participants with presence and absence of anxiety/depression was assessed with the area under the receiver operating characteristic (ROC) curve. The area under the curve (AUC) of > 0.7 was considered as acceptable fit. Statistical significance for all analyses was set to *p* <  0.05.

## Results

### Demographic, social, and clinical characteristics, personality traits, and quality of life

The demographic, social, and clinical characteristics of participants, as well as their personality traits and quality of life, are summarized in Table 1. A total of 300 participants were enrolled in the study. The median age of the participants was 63 years (IQR = 16 years). Approximately half of the participants were males (*n* = 158, 52.7%). More than half of the participants were Malays (*n* = 195, 65.0%) and a similar proportion of participants were Muslim (*n* = 199, 66.3%). Large proportion of the participants also agreed that they had regular religious practice (*n* = 237, 79.0%). The majority of participants were married (*n* = 233, 77.7%), and an almost equal proportion of participants received up to a secondary level of education (*n* = 133, 44.3%) or a tertiary level of education (*n* = 119, 39.7%). A minority of participants were retired (*n* = 123, 41%), and slightly more than half were earning less than RM 3000 per month (*n* = 166, 55.3%). The majority of participants perceived that they had good social support, with 80.3% (*n* = 241) of participants ‘agreeing’ with adequate social support. The majority of participants had never smoked (*n* = 216, 72.0%), had never consumed alcohol (*n* = 271, 90.3%), and had no history of recreational drug use (*n* = 294, 98%).

**Table 1:** Socio-demographic, social and clinical characteristics of the participants

Variables	N	%
Age (*N* = 300)	63^a^	16^b^
Gender		
Male	158	52.7
Female	141	47.0
Missing	1	0.3
Ethnicity		
Malay	195	65.0
Chinese	54	18.0
Indian	44	14.7
Others	7	2.3
Marital status		
Married	233	77.7
Single	24	8.0
Divorced/widowed	43	14.3
Education		
None	7	2.3
Primary	38	12.7
Secondary	133	44.3
Tertiary	119	39.7
Missing	3	1.0
Employment		
Employed	80	26.7
Unemployed	94	31.3
Retired	123	41.0
Missing	3	1.0
Household income		
< RM3,000	166	55.3
RM3,000–6000	57	19.0
> RM6,000	61	20.3
Missing	16	5.3
Religion		
Islam	199	66.3
Buddhism	37	12.3
Hindusim	35	11.7
Christianity	23	7.7
Others	4	1.3
Missing	2	0.7
Regular religious practice		
Disagree	19	6.3
Neutral	43	14.4
Agree	237	79.0
Missing	1	0.3
Smoking		
Never	216	72.0
Ex-smoker	64	21.3
Current smoker	20	6.7
Alcohol use		
Yes	26	8.7
No	271	90.3
Missing	3	1.0
Recreational drug use		
Yes	5	1.7
No	294	98.0
Missing	1	0.3
Perceived social support		
Very poor	2	0.7
Poor	8	2.7
Neutral	48	16.0
Good	165	55.0
Very good	76	25.3
Missing	1	0.3
Diabetes type		
Type 1	22	7.3
Type 2	269	89.7
Gestational	6	2.0
Missing	3	1.0
Duration of DM (years) (*N* = 229)	14^a^	12^b^
Insulin therapy		
Yes	138	46.0
No	114	38.0
Missing	48	16.0
HbA1c (%) (*N* = 268)	7.6^a^	2.7^b^
Diabetic control		
Good	92	30.7
Poor	208	69.3
“I am able to manage my diabetes well”		
Disagree	15	5.0
Neutral	70	23.4
Agree	214	71.3
Missing	1	0.3
Obesity		
BMI < 25	65	21.7
BMI 25–30	147	49.0
BMI > 30	78	26.0
Missing	10	3.3
Hypertension		
Yes	222	74.0
No	78	26.0
Dyslipidaemia		
Yes	152	50.7
No	148	49.3
Ischaemic heart disease		
Yes	82	27.3
No	218	72.7
Stroke		
Yes	27	9.0
No	273	91.0
Renal disease		
Yes	53	17.7
No	247	82.3
Anxiety (GAD-7)		
Yes	27	9.0
No	273	91.0
Depression (BDI)		
No/minimal	240	80.0
Mild to moderate	41	13.7
Moderate to severe	17	5.7
Severe	2	0.7
WHOQOL-BREF		
Overall perception of QOL		
Very poor	3	1.0
Poor	5	1.7
Neither poor nor good	89	29.7
Good	162	54.0
Very good	41	13.7
Overall perception of health		
Very dissatisfied	1	0.3
Dissatisfied	37	12.3
Neither satisfied nor dissatisfied	131	43.7
Satisfied	121	40.3
Very satisfied	10	3.3
WHOQOL-BREF domains (N = 300)		
Physical health	63.00^a^	19.00^b^
Psychological	69.00^a^	13.53^b^
Social relationships	75.00^a^	19.00^b^
Environment	69.00^a^	12.00^b^
BFI subscales (N = 300)		
Extraversion	3.38^a^	0.75^b^
Agreeableness	3.78^a^	0.43^b^
Conscienciousness	3.67^a^	0.60^b^
Neuroticism	2.50^a^	0.73^b^
Openness	3.30^a^	0.60^b^

^a^ = Median; ^b^ = Interquartile range (IQR)

With regard to clinical characteristics, the majority of participants were diagnosed with type 2 diabetes mellitus (*n* = 269, 89.7%), while a minority had been diagnosed with type 1 diabetes mellitus (*n* = 22, 7.3%) or gestational diabetes mellitus (*n* = 6, 2.0%). The median duration of diabetes diagnosis was 14 years (IQR = 12 years), while the median HbA1_C_ measurement was 7.6% (IQR = 2.7%). Almost half of the participants were on insulin therapy (*n* = 138, 46.0%). More than half of the participants perceived that they managed their illness well; in fact, 71.3% (*n* = 214) of participants ‘agreed’ with the statement ‘I am able to manage my diabetes well’. Almost half (49.0%, *n* = 147) of participants were overweight (BMI 25–30), while 26.0% (*n* = 78) were obese (BMI > 30).

Screening with the GAD-7 indicated that only a small proportion of the participants had anxiety (9%, *n* = 27), while BDI-II screening revealed that a relatively larger proportion of participants had depression (20%, *n* = 60). In BFI assessment, the median of extraversion was 3.38 (IQR = 0.75), agreeableness was 3.78 (IQR = 0.43), conscientiousness was 3.67 (IQR = 0.60), neuroticism was 2.50 (IQR = 0.73), and openness was 3.30 (IQR = 0.60). The WHOQOL-BREF screening revealed that the median of the physical health score was 14.29 (IQR = 3.43), the psychological score was 15.33 (IQR = 2.67), the social relationships score was 16.00 (IQR = 2.67), and the environment score was 15.00 (IQR = 2.50). The mean and standard deviation (SD) of the BFI subscale scores and the WHOQOL-BREF domain scores are summarized in Table S1 in Additional file 1 (supplementary information).

### The association between demographic, social, and clinical characteristics, personality traits and quality of life, and anxiety among participants

The findings of the univariate logistic regression analyses examining associations between demographic, social, and clinical characteristics, personality traits, quality of life, and anxiety are summarized in Table 2. The demographic characteristics associated with anxiety (*p* <  0.25) were ethnicity, employment status, household income, and regular religious practice. There were no significant association between history of cigarette smoking, alcohol intake, and recreational drug use, and anxiety. On the contrary, there were several clinical characteristics, personality traits, and quality of life components which were associated with anxiety. The variables which were associated with anxiety include self-perceived diabetic management, depression, overall perception of QOL, overall perception of health, physical quality of life, psychological quality of life, social quality of life, environmental quality of life, extraversion, agreeableness, conscientiousness, neuroticism, and openness scores, and the interaction between perceived social support and neuroticism.

**Table 2:** The association between individual socio-demographic, social and clinical characteristics, and anxiety among participants

Variables	Crude OR (95% CI)	*p*-value
Age	0.99 (0.96–1.02)	0.585
Gender		
Male	1	
Female	0 .75 (0 .34–1.68)	0.485
Ethnicity:		
Non-Malays	1	
Malays	0 .39 (0.18–0.88)	0.022*
Marital status:		
Married	1	
Not married	1.73 (0.58–5.17)	0.331
Education:		
Secondary & below	1	
Tertiary	1.05 (0.47–2.35)	0.905
Employment:		
Employed	1	
Unemployed	0.53 (0.20–1.36)	0.186*
Retired	0.34 (0.13–0.91)	0.032*
Household income:		
< RM3,000	1	
RM3,000–6000	0.41 (0.12–1.42)	0.158*
> RM6,000	0.38 (0.11–1.32)	0.127*
Regular religious practice:		
Disagree	1	
Neutral	0.18 (0.03–1.06)	0.058*
Agree	0.34 (0.10–1.11)	0.074*
Perceived social support:		
Poor	1	
Neutral	1.05 (0.19–5.76)	0.953
Good	0.26 (0.05–1.36)	0.311
Cigarette smoking:		
Non-smoker	1	
Smoker	1.58 (0.69–3.61)	0.276
Alcohol:		
No	1	
Yes	0.38 (0.05–2.93)	0.354
Recreational drug use:		
No	1	
Yes	2.59 (0.28–24.01)	0.403
Obesity:		
BMI < 25	1	
BMI 25–30	1.99 (0.64–6.14)	0.254
BMI > 30	1.33 (0.36–4.94)	0.667
Diabetes mellitus type:		
Type I or gestational diabetes	1	
Type II diabetes	2.74 (0.36–21.00)	0.333
Insulin therapy:		
No	1	
Yes	1.51 (0.64–3.57)	0.350
Good self-perceived diabetic management:		
Disagree	1	
Neutral	0.19 (0.05–0.69)	0.011*
Agree	0.10 (0.03–0.31)	< 0.001*
Diabetic control:		
Good	1	
Poor	1.61 (0.63–4.13)	0.322
Depression:		
No depression	1	
Depression	36.68 (12.00–112.06)	< 0.001*
Overall perception of QOL:		
Poor/very poor	1	
Neutral	0.10 (0.02–0.48)	0.004*
Good/very good	0.03 (0.01–0.14)	< 0.001*
Overall perception of health		
Poor/very poor	1	
Neutral	0.18 (0.07–0.46)	< 0.001*
Good/very good	0.09 (0.03–0.27)	< 0.001*
WHOQOL-BREF domains		
Physical health	0.72 (0.61–0.85)	< 0.001*
Psychological	0.48 (0.38–0.62)	< 0.001*
Social relationships	0.61 (0.51–0.74)	< 0.001*
Environment	0.60 (0.48–0.56)	< 0.001*
BFI subscales		
Extraversion	0.38 (0.17–0.85)	0.018*
Agreeableness	0.20 (0.07–0.54)	0.001*
Conscientiousness	0.52 (0.22–1.23)	0.135*
Neuroticism	16.84 (6.16–40.01)	< 0.001*
Openness	1.91 (0.85–4.29)	0.119*
Neuroticism x perceived social support	1.24 (1.06–1.46)	0.009*

^*^p-value < 0.25

### The association between demographic, social, and clinical characteristics, personality traits and quality of life, and depression among participants

The findings of the univariate logistic regression analyses examining the associations between demographic, social, and clinical characteristics, personality traits, quality of life, and depression among participants are summarized in Table 3. There were four demographic characteristics associated with depression (*p* <  0.25), such as age, employment status, household income, and regular religious practice. There were no significant associations between social characteristics and depression among participants. Several clinical characteristics, personality traits, and quality of life components were associated with depression. The variables which were associated with depression include self-perceived diabetic management, anxiety, overall perception of QOL, overall perception of health, physical quality of life, psychological quality of life, social quality of life, environmental quality of life, extraversion, agreeableness, conscientiousness, and neuroticism scores, and the interaction between perceived social support and neuroticism.

**Table 3:** The association between individual socio-demographic, social and clinical characteristics, and depression among participants

Variables	Crude OR (95% CI)	*p*-value
Age	0.98 (0.96–0.99)	0.030*
Gender		
Male	1	
Female	0.76 (0.43–1.34)	0.341
Ethnicity		
Non-Malays	1	
Malays	0.84 (0.47–1.50)	0.545
Marital status:		
Married	1	
Not married	1.05 (0.53–2.08)	0.890
Education		
Secondary & below	1	
Tertiary	0.85 (0.48–1.53)	0.596
Employment		
Employed	1	
Unemployed	0.91 (0.46–1.81)	0.788
Retired	0.42 (0.20–0.87)	0.019*
Household income		
< RM3,000	1	
RM3,000–6000	0.96 (0.41–1.97)	0.916
> RM6,000	0.36 (0.14–0.89)	0.027*
Regular religious practice		
Disagree	1	
Neutral	0.24 (0.07–0.78)	0.018*
Agree	0.22 (0.08–0.58)	0.002*
Perceived social support		
Poor	1	
Neutral	2.40 (0.46–12.57)	0.300
Good	0.79 (0.16–3.87)	0.773
Cigarette smoking		
Non-smoker	1	
Smoker	1.13 (0.61–2.10)	0.700
Alcohol		
No	1	
Yes	0.50 (0.14–1.71)	0.268
Recreational drug use		
No	1	
Yes	0.98 (0.11–9.10)	0.988
Obesity		
BMI < 25	1	
BMI 25–30	1.32 (0.64–2.75)	0.454
BMI > 30	1.02 (0.44–2.39)	0.962
Diabetes mellitus type		
Type I or gestational diabetes	1	
Type II diabetes	1.11 (0.40–3.06)	0.840
Insulin therapy:		
No	1	
Yes	0.83 (0.46–1.47)	0.513
Good self-perceived diabetic management		
Disagree	1	
Neutral	0.49 (0.16–1.53)	0.218*
Agree	0.20 (0.07–0.59)	0.004*
Diabetic control		
Good	1	
Poor	1.42 (0.74–2.70)	0.289
Anxiety		
No Anxiety	1	
Anxiety	36.68 (12.00–112.06)	< 0.001*
Overall perception of QOL		
Poor/very poor	1	
Neutral	0.14 (0.03–0.73)	0.019*
Good/very good	0.05 (0.01–0.28)	< 0.001*
Overall perception of health		
Poor/very poor	1	
Neutral	0.46 (0.21–0.98)	< 0.045*
Good/very good	0.20 (0.09–0.46)	< 0.001*
WHOQOL-BREF domains		
Physical health	0.66 (0.58–0.76)	< 0.001*
Psychological	0.58 (0.49–0.61)	< 0.001*
Social relationships	0.68 (0.59–0.77)	< 0.001*
Environment	0.66 (0.56–0.78)	< 0.001*
BFI subscales		
Extraversion	0.50 (0.28–0.89)	0.018*
Agreeableness	0.36 (0.18–0.73)	0.004*
Conscientiousness	0.33 (0.17–0.63)	0.001*
Neuroticism	5.54 (3.02–10.18)	< 0.001*
Openness	1.23 (0.70–2.17)	0.476
Neuroticism x perceived social support	1.19 (1.06–1.33)	0.004*

* *p*-value < 0.25

### Stepwise multiple logistic regression analyses between various factors and anxiety among participants

The findings of stepwise multiple logistic regression analyses between demographic characteristics (ethnicity, employment, household income, and practice of religion), clinical factors (co-morbid depression), personality traits (extraversion, agreeableness, neuroticism, conscientiousness, openness, and the interaction between perceived social support and neuroticism), quality of life (overall perception of QOL, overall perception of health, physical, psychological, social, and environmental QOL), and anxiety among participants are summarized in Table 4. There were only a few factors predictive of anxiety among participants. Participants who were depressed (adjusted OR = 9.89, 95% CI = 2.63–37.14, *p* = 0.001) with higher neuroticism scores (adjusted OR = 11.66, 95% CI = 2.69–50.47, *p* = 0.001) had higher odds of having anxiety. On the contrary, lower odds of anxiety was associated with higher psychological scores on the quality of life questionnaire (adjusted OR = 0.47, 95% CI = 0.29–0.75, *p* = 0.002) and higher conscientiousness scores (adjusted OR = 0.45, 95% CI = 0.23–0.80, *p* = 0.004). Other demographic characteristics, personality traits, and QOL components were not significant predictors of occurrence of anxiety among the participants. The logistic regression model reported a Cox and Snell R^2^ of 0.29 (*p* <  0.001), Hosmer-Lemeshow goodness-of-fit test was not significant (*p* = 0.843) and the area under the ROC curve (AUC) of 0.949 (95% CI = 0.912–0.986, p <  0.001), indicating acceptable fit of the model to discriminate participants with presence and absence of anxiety.

**Table 4:** Stepwise multiple logistic regression model between various factors and anxiety among participants

Variables	Adjusted OR^a^ (95% CI)	*p*-value
Depression		
No depression	1	
Depression	9.89 (2.63–37.14)	0.001*
Psychological domain of WHOQOL-BREF	0.47 (0.29–0.75)	0.002*
Conscientiousness	0.45 (0.23–0.80)	0.004*
Neuroticism	11.66 (2.69–50.47)	0.001*

*Statistical significance at *p* <  0.05, ^a^ The stepwise logistic regression model indicated ethnicity, employment, household income, practice of religion, perceived diabetic management, overall perception of QOL, overall perception of health, physical, social relationship and environment domains of QOL, extraversion, agreeableness, openness personality traits, and neuroticism x perceived social support were not significantly associated with anxiety among the participants. The model reported reported Cox and Snell R^2^ = 0.29, *p* <  0.001, Hosmer-Lemeshow goodness-of-fit test (*p* = 0.843), and the area under the receiver operating characteristic curve (AUC) = 0.949 (95% CI = 0.912–0.986, *p* < 0.001)

### Stepwise multivariate logistic regression analyses between various factors and depression among participants

The findings of stepwise multivariate logistic regression analyses between demographic characteristics (age, employment, household income, and practice of religion), clinical factors (co-morbid anxiety and perceived diabetic management), personality traits (extraversion, agreeableness, conscientiousness, neuroticism, and the interaction between perceived social support and neuroticism), quality of life (overall perception of QOL and health, physical, psychological, social, and environmental QOL), and depression among participants are summarized in Table 5. The only clinical factor associated with higher odds of depression was anxiety, which increased the occurrence of depression by almost 20-fold (adjusted OR = 19.83, 95% CI = 5.63–69.92, *p* <  0.001). On the contrary, older age (adjusted OR = 0.96, 95% CI = 0.93–0.98, *p* = 0.002), higher physical health quality of life scores (adjusted OR = 0.69, 95% CI = 0.58–0.83, *p* <  0.001), and higher social quality of life scores (adjusted OR = 0.84, 95% CI = 0.71–.0.99, *p* = 0.047) were associated with lower odds of occurrence of depression. Perceived diabetic management, other demographic characteristics, personality traits, and QOL components did not significantly predict depression among the participants. The logistic regression model reported a Cox and Snell R^2^ of 0.294 (*p* <  0.001), Hosmer-Lemeshow goodness-of-fit test was not significant (*p* = 0.447) and the area under the ROC curve (AUC) of 0.851 (95% CI = 0.793–0.909, *p* <  0.001), indicating acceptable fit of the model to discriminate participants with presence and absence of depression.

**Table 5:** Stepwise multiple logistic regression model between various factors and depression among participants

Variables	Adjusted OR^a^ (95% CI)	*p*-value
Age	0.96 (0.93–0.98)	0.002*
Anxiety:		
No	1	
Yes	19.83 (5.63–69.92)	< 0.001*
Physical domain of WHOQOL-BREF	0.69 (0.58–0.83)	< 0.001*
Social relationship domain of WHOQOL-		
BREF	0.84 (0.71–.0.99)	0.047*

*Statistical significance at *p* < 0.05, ^a^ The stepwise logistic regression model indicated employment, household income, practice of religion, perceived diabetic management, overall perception of QOL, overall perception of health, psychological and environment domains of QOL, extraversion, agreeableness, conscientiousness, neuroticism personality traits, and neuroticism x perceived social support were not significantly associated with depression among the participants. The model reported reported Cox and Snell *R*^2^ = 0.294, *p* < 0.001, Hosmer-Lemeshow goodness-of-fit test (*p* = 0.447), and the area under the receiver operating characteristic curve (AUC) = 0.851 (95% CI = 0.793–0.909, *p* < 0.001)

## Discussion

The ADAPT-DM study aimed to determine the prevalence of depression and anxiety, and their associated factors among Malaysian patients with diabetes. Regarding the prevalence of depression and anxiety, we found that 9% of the participants screened positive for anxiety and 20% met criteria for depression. The prevalence of depression reported in our study was similar to that reported in previous studies, where prevalence was estimated between 18 and 30% [25]. The prevalence of anxiety among participants in our study was relatively low compared to the prevalence reported by the INTERPRET-DD study, which estimated the prevalence of anxiety (all anxiety disorder included) to be 18% based on data collected from 3170 diabetic patients from 15 countries in different continents [26]. This may be explained by the difference in instruments used for assessing anxiety. While we used the GAD-7, which is designed to assess for generalized anxiety disorder (GAD), the INTERPRET-DD study used the Mini International Neuropsychiatric Interview. The prevalence of GAD specifically reported by the INTERPRET-DD study was 8.1%, which was similar to the prevalence of anxiety disorder reported in our study [26].

Our findings reveal that neuroticism and depression increased the odds of developing anxiety by almost 12-fold and 10-fold respectively. Better psychological QOL and higher conscientiousness were protective against anxiety which reduced the occurrence of anxiety by half (0.47-fold and 0.45-fold respectively). The occurrence of depression greatly increased the odds of anxiety in our study, which is similar to what was reported in a study of 893 Chinese patients with diabetes [27]. The positive correlation between depression and anxiety is well documented in chronic illness, and the occurrence of depression can increase the risk of anxiety symptoms in patients with chronic illness [28, 29]. This relationship is expected as some theories suggested that anxiety and depression shared the same neurobiological mechanism in which they represent different phenotypic manifestations which run in a continuum [30]. The association between neuroticism and anxiety disorders, particularly generalized anxiety disorder and panic disorder, is well documented in the general population [31]. People with trait neuroticism tend to utilize maladaptive forms of emotional regulation rather than reappraisal which is believed to increase the severity of anxiety symptoms in these individuals [32]. However, higher psychological QOL reduced the odds of anxiety disorders in diabetic patients, which is in line with the findings of other studies on patients with diabetes [33, 34]. As expected, conscientiousness is inversely related to anxiety disorders, such as generalized anxiety disorder, panic disorder, agoraphobia, and social phobia [35]. A person with high conscientiousness possess traits, such as orderliness, high self-efficacy, achievement-driven, self-discipline, and responsible. Hence, a person with high conscientiousness tend to find stressful situations to be less demanding as they utilise adaptive coping strategies to overcome the effects of stressful situations, such as effective cognitive restructuring and instrumental problem solving. As a result, these traits in a person with high conscientiousness may help to reduce anxiety [32].

Our study indicated that among demographic and social characteristics, older age reduced odds of depression by 0.96-fold. Regarding clinical characteristics, those with anxiety had almost 20-fold increased odds of developing depression. Greater physical health-related QOL and higher social relationship-related QOL reduced the occurrence of depression by 0.69-fold and 0.84-fold respectively. Several studies have suggested that spirituality and religiosity are protective factors against depression, particularly in the elderly [36–38]. In our sample, 79% of participants within the elderly age group (age of > 60 years old) reported having a strong religious practice. Although religious practice was not a significant predictor of depression in our study when all the participants with various age groups were taken into consideration, a large proportion of elderly participants reported having a strong religious practice and the fact that increasing age reduced the odds of depression, may indicate that strong religious practice could mediate the protective effect of older age against depression in our study. A bidirectional relationship between mood disorders and diabetes has been proposed, and the occurrence of anxiety is known to increase the risk of developing depression among patients [28, 29]. Our findings further support a bidirectional association between anxiety and depression among patients with diabetes, as reported by previous studies [9, 27]. We found that greater physical health-related QOL acts as a protective factor to reduce the odds of depression in diabetic patients. Similar results were found in a systematic review of 20 studies of diabetic patients across Europe and the United States [39]. Social support moderates the effect of stress on depression in which the impact of stress on depression is small in people with high social support, particularly those with high perceived spousal support [40, 41]. This may well explain the reason why high social relationship-related QOL was protective against depression in our study. Regarding personality traits, unlike previous studies in the general population, our findings did not suggest a predictive effect of neuroticism on depression in patients with diabetes. This discrepancy may be explained by the moderating effect of social support on the association between neuroticism and depression. Environmental factor could be a mediating or moderating factor in the relationship between personality traits and depression [42]. Although perceived social support was not a significant predictor of depression in this study, it may reduce the effect of neuroticism on depression. Large proportion of participants reported strong social support (80.3%) in this study. High social support may reduce the effect of neuroticism on depression as indicated by a lower odds ratio of the interaction between perceived social support and neuroticism on depression (crude OR = 1.19) as compared to the odds ratio of neuroticism alone on depression (crude OR = 5.54) in univariate regression analysis (Table 3). In the multiple regression model, neither the interaction between perceived social support and neuroticism nor neuroticism alone significantly predicted depression (Table 5).

The current study should be considered in light of its limitations. First, this study was conducted in a single tertiary healthcare referral centre. Hence, the findings may not be generalizable to the entire diabetic population in the country. Second, the cross-sectional design of the study does not allow determination of the causal relationship between the associated factors, depression and anxiety. Third, the depressive and anxiety symptoms were measured by self-reported tools rather than diagnostic interviews, which may affect the reliability of participant classification into the depressive and anxiety groups. Fourth, chronic pain is a common symptom among patients with diabetes [43], and it often coexists and interacts with anxiety and depression in these individuals [44]. However, we did not measure chronic pain as one of the predicting factors in our study, although it could be an important confounding factor which may contribute to depression and anxiety among diabetic patients.

Despite these limitations, the study had many strengths. The data obtained included a wide range of factors that could potentially be associated with depression and anxiety in diabetic patients. The study sample demonstrated diagnostic heterogeneity (patients with type 1, type 2, and gestational diabetes were included), representative of the Malaysian diabetic population. Our study examined the association between personality traits, quality of life, depression, and anxiety in patients with diabetes, which has previously been poorly characterized. Our study highlights a need to screen not only for psychiatric complications of diabetes, such as depression and anxiety, but also personality traits and quality of life. Hence, management of diabetes mellitus requires a multidisciplinary team that can manage both physical and mental health of patients.

## Conclusions

The ADAPT-DM study reported a relatively lower prevalence of anxiety and similar prevalence of depression in a large and heterogeneous sample of Malaysian diabetic patients as compared to studies in other countries. Co-morbid depression and higher neuroticism increased odds of developing anxiety. Greater psychological QOL and higher conscientiousness were protective against occurrence of anxiety. Co-morbid anxiety increased the odds of developing depression, while older age, greater physical health-related QOL, and higher social relationship-related QOL were protective against depression. Our findings indicate that screening for personality traits and QOL are necessary to manage anxiety and depression for a holistic approach of diabetic treatment.

## Supplementary information

**Additional file 1 Table S1.** The mean and standard deviation for age, the WHOQOL-BREF, and the BFI scores.

## Data Availability

The datasets used and/or analysed during the current study are available from the corresponding author upon reasonable request.

## Abbreviations

QOL: Quality of life
BMI: Body mass index
GAD: Generalized anxiety disorder
IQR: Interquartile range
OR: Odds ratio
95% CI: 95% confidence interval
ADAPT-DM: Anxiety, Depression, and Personality Traits in Diabetes Mellitus
UKMMC: Universiti Kebangsaan Malaysia Medical Centre
GAD-7: Seven-item Generalised Anxiety Disorder scale
BDI: Beck Depression Inventory-II
BFI: Big Five Inventory
WHOQOL-BREF: World Health Organization Quality of Life-BREF
ROC curve: Receiver operating characteristic curve
AUC: Area under the receiver operating characteristic curve
