# Supplementary material for: Depression, anxiety, and associated factors in patients with diabetes: evidence from the anxiety, depression, and personality traits in diabetes mellitus (ADAPT-DM) study
Source: BMC Psychiatry. 2020 May 12;20:227. doi: 10.1186/s12888-020-02615-y (PMC7218550; doi:10.1186/s12888-020-02615-y)
Supplement: Supplementary file 1 — Additional file 1 Table S1. The mean and standard deviation for age, the WHOQOL-BREF, and the BFI scores. [file 12888_2020_2615_MOESM1_ESM.docx]

Table S1. The mean and standard deviation for age, the WHOQOL-BREF, and the BFI scores

| Variables | Mean | Standard deviation (SD) |
| --- | --- | --- |
| Age (in years) (N=300):  WHOQOL-BREF domains (N=300):  Physical health  Psychological  Social relationships  Environment  BFI subscales (N=300):  Extraversion  Agreeableness  Conscientiousness  Neuroticism  Openness | 60.38  63.34  69.87  69.05  70.74  3.40  3.83  3.67  2.47  3.26 | 13.39  15.47  13.53  16.31  13.21  0.52  0.43  0.47  0.57  0.50 |
